# Minimal important difference of the 12-item World Health Organization Disability Assessment Schedule (WHODAS) 2.0 in persons with chronic low back pain

**DOI:** 10.1186/s12998-023-00521-0

**Published:** 2023-12-05

**Authors:** Jessica J. Wong, Sheilah Hogg-Johnson, Wouter De Groote, Agnieszka Ćwirlej-Sozańska, Olatz Garin, Montse Ferrer, Àngels Pont Acuña, Pierre Côté

**Affiliations:** 1 Institute for Disability and Rehabilitation Research, Faculty of Health Sciences, Ontario Tech University, 2000 Simcoe Street North, Oshawa, ON L1G 0C5 Canada; 2 Graduate Studies, Canadian Memorial Chiropractic College, 6100 Leslie Street, Toronto, ON M2H 3J1 Canada; 3 Department of Research and Innovation, Canadian Memorial Chiropractic College, 6100 Leslie Street, Toronto, ON M2H 3J1 Canada; 4 Dalla Lana School of Public Health, University of Toronto, 155 College St Room 500, Toronto, ON M5T 3M7 Canada; 5 Rehabilitation Programme, Department of Noncommunicable Diseases, Sensory Functions, Disability, and Rehabilitation Unit, World Health Organization, Avenue Appia 20, Geneva, 1211 Switzerland; 6 Institute of Health Sciences, College of Medical Sciences, University of Rzeszow, Rejtana Street 16C, Rzeszow, 35-959 Poland; 7 Laboratory of Geronto-prophylaxis, Center for Innovative Research in Medical and Natural Sciences, Rzeszow of University, Warzywna Street 1A, Rzeszow, 35-310 Poland; 8 Health Services Research Unit, IMIM-Hospital del Mar, C/ del Dr. Aiguader, 88, Barcelona, 08003 Spain; 9 CIBER en Epidemiología y Salud Pública (CIBERESP), Av. Monforte de Lemos, 3-5. Pabellón 11. Planta 0, Madrid, 28029 Spain; 10 Universitat Autònoma de Barcelona, Ronda de la Univ, Barcelona, 21, 08007 Spain

**Keywords:** World Health Organization Disability Assessment Schedule, Low back pain, Minimal important difference, Functioning, Disability, Measurement

## Abstract

**Background:**

The World Health Organization Disability Assessment Schedule 2.0 12-item survey (WHODAS-12) is a questionnaire developed by the WHO to measure functioning across health conditions, cultures, and settings. WHODAS-12 consists of a subset of the 36 items of WHODAS-2.0 36-item questionnaire. Little is known about the minimal important difference (MID) of WHODAS-12 in persons with chronic low back pain (LBP), which would be useful to determine whether rehabilitation improves functioning to an extent that is meaningful for people experiencing the condition. Our objective was to estimate an anchor-based MID for WHODAS-12 questionnaire in persons with chronic LBP.

**Methods:**

We analyzed data from two cohort studies (identified in our previous systematic review) conducted in Europe that measured functioning using the WHODAS-36 in adults with chronic LBP. Eligible participants were adults with chronic LBP with scores on another measure as an anchor to indicate participants with small but important changes in functioning over time [Short-form-36 Physical Functioning (SF36-PF) or Oswestry Disability Index (ODI)] at baseline and follow-up (study 1: 3-months post-treatment; study 2: 1-month post-discharge from hospital). WHODAS-12 scores were constructed as sums of the 12 items (scored 0–4), with possible scores ranging from 0 to 48. We calculated the mean WHODAS-12 score in participants who achieved a small but meaningful improvement on SF36-PF or ODI at follow-up. A meaningful improvement was an MID of 4–16 on ODI or 5–16 on SF36-PF.

**Results:**

Of 70 eligible participants in study 1 (mean age = 54.1 years, SD = 14.7; 69% female), 18 achieved a small meaningful improvement based on SF-36 PF. Corresponding mean WHODAS-12 change score was − 3.22/48 (95% CI -4.79 to -1.64). Of 89 eligible participants in study 2 (mean age = 65.5 years, SD = 11.5; 61% female), 50 achieved a small meaningful improvement based on ODI. Corresponding mean WHODAS-12 change score was − 5.99/48 (95% CI − 7.20 to -4.79).

**Conclusions:**

Using an anchor-based approach, the MID of WHODAS-12 is estimated at -3.22 (95% CI -4.79 to -1.64) or -5.99 (95% CI − 7.20 to -4.79) in adults with chronic LBP. These MID values inform the utility of WHODAS-12 in measuring functioning to determine whether rehabilitation or other health services achieve a minimal difference that is meaningful to patients with chronic LBP.

**Supplementary Information:**

The online version contains supplementary material available at 10.1186/s12998-023-00521-0.

## Introduction

At least one in three people globally will require rehabilitation at some point in their life [1], and rehabilitation needs will increase over time [2]. However, this increasing need for rehabilitation is largely unmet [2]; consequently, the World Health Organization (WHO) issued a call to increase access to rehabilitation services globally through strengthening health systems for rehabilitation [3]. Low back pain (LBP) is the main reason for unmet rehabilitation needs globally [1, 4]. It is thus critically important that people with LBP receive rehabilitation services to improve functioning and health outcomes.

To understand the utility of rehabilitation care, it is important to measure whether the delivery of rehabilitation services effectively improves functioning at individual and population levels. WHO Disability Assessment Schedule 2.0 (WHODAS) is a self-reported questionnaire developed by the WHO as a generic tool that integrates an individual’s level of functioning in major life domains, directly linked to the International Classification of Functioning, Disability and Health [5]. WHODAS is applicable across various cultures and settings, and easy to administer in clinical and population-based settings [5]. To assess whether rehabilitation is effective, it is useful to determine whether receiving rehabilitation services achieves the minimal important difference (MID). However, little is known on the MID of WHODAS-12 in persons with chronic LBP.

We conducted a systematic review [6] on the psychometric properties of the WHODAS and identified one study reporting MIDs for the WHODAS-12 in patients with musculoskeletal conditions [7]. Specifically, in patients with chronic musculoskeletal pain (including LBP) in Finland, MID of WHODAS-12 was estimated as a range of 3.09 to 4.68 out of 48 using distribution-based methods [7]. More studies are needed to estimate the MID of WHODAS-12 in persons with chronic LBP, particularly using anchor-based methods by considering important differences in other outcome measures (e.g., global perceived recovery) to facilitate triangulation from multiple anchors/methods. COnsensus-based Standards for the selection of health Measurement Instruments (COSMIN) recommends using an anchor-based longitudinal approach to determine MID to reflect what patients consider important, rather than distribution-based methods which often uses standard deviation as the metric related to pre-treatment variability in the measure [8].

Our systematic review [6] identified two longitudinal studies that examined the measurement properties of WHODAS-36 in persons with chronic LBP [9, 10]. Since specific WHODAS-36 questions can be used to compute WHODAS-12 scores, we proposed secondary use of data from these two original studies to estimate the MID of WHODAS-12 in persons with chronic LBP. Therefore, our objective was to compute an anchor-based MID for the WHODAS-12 questionnaire in persons with chronic LBP.

## Methods

We analyzed data from two cohort studies that measured functioning using WHODAS-36 in adults with chronic LBP [9, 10] at two points in time. This project has been approved by the Research Ethics Board at Ontario Tech University (Reference #17173).

We selected these two studies based on our previous systematic review [6]. Our systematic review examined the measurement properties and minimal important difference of the 36-item and 12-item WHODAS questionnaire in persons with LBP. This systematic review identified only one cross-sectional study that estimated the MID of WHODAS-12 in this population using distribution-based methods. The systematic review also identified two longitudinal studies with WHODAS-36 data that could convert to WHODAS-12 scores and estimate MID using an anchor-based approach, which are the two studies included in this analysis [9, 10]. Based on critical appraisal using COSMIN and COSMIN-OMERACT checklists in the systematic review, the study by Cwirlej-Sozanska et al. was deemed very good for internal consistency, adequate for reliability, doubtful for construct validity, and doubtful for responsiveness; the study by Garin et al. was deemed doubtful for construct validity [6].

### Study sample

Eligible participants were adults with chronic LBP who completed WHODAS and another measure that could be used as an anchor to identify subjects experiencing a small but important change in functioning between the two measurement points. The anchor measures that we used were the Short-form-36 Physical Functioning dimension (SF-36 PF) in study 1 [9] and Oswestry Disability Index (ODI) in study 2 [10]. These were selected as they measure closely related constructs to that of the WHODAS-12. Study 1 by Garin et al. included adults aged ≥ 18 years with different chronic conditions recruited from seven European Centres in Czech Republic, Germany, Italy, Slovenia, and Spain [9]. Chronic LBP was defined as ≥ 12 weeks’ duration in this study. The original sample in study 1 had a mean age of 52.7 years (SD 15.6), 56.2% were female, and the mean score on the 36-item WHODAS was 24.8 (SD 19.3); 9.9% of the entire sample had LBP. Evaluations were made at baseline (pre-treatment), six weeks, and three months. For our study, we restricted to participants with chronic LBP and focused on data from baseline and 3-month follow-up, as 3-month follow-up was originally intended to assess responsiveness of WHODAS-36. Study 2 by Cwirlej-Sozanska et al. included patients (aged ≥ 50 years) with chronic LBP (≥ 12 weeks’ duration) admitted to the rehabilitation ward of a family specialist hospital in Poland [10]. Exclusion criteria were severe neurological disorders of the central nervous system (stroke and traumatic brain injury), unstable cardiovascular diseases, active cancer, and amputations. The original sample in study 2 had a mean age of 66 years (SD 11.6), 62.0% were female, and mean score on the 36-item WHODAS was 41.5 (SD 13.8). Evaluations were at baseline (admission), two days post-admission, and one month after completion of rehabilitation in the hospital. For our study, we focused on data from baseline and 1-month post-discharge from hospital to compute an MID for WHODAS-12. Further details of each study are described in the original articles [9, 10].

### WHO Disability Assessment Schedule

WHODAS 2.0 is a generic, self-reported assessment instrument developed by the WHO to provide a standardized method for measuring functioning across health conditions, cultures, and settings [5]. The short version of the WHODAS 2.0, WHODAS-12, has 12 questions rated from 0 (no difficulty) to 4 (extreme difficulty/cannot do), which are a subset of the 36 questions from the full version (WHODAS-36) (see Additional File 1) [5], with two questions from each of the six domains: (1) Cognition (items 3, 6); (2) Mobility (items 1, 7); (3) Self-care (items 8, 9); (4) Getting along (items 10, 11); (5) Life activities (items 2, 12); and (6) Participation (items 4, 5) [5]. Since the original data from studies 1 and 2 had WHODAS-36 questions and scores, the WHODAS-12 could be constructed from the specific WHODAS-12 questions. Simple scoring involves adding up the scores from each WHODAS-12 item to compute a summary score out of 48 (higher scores mean greater limitations in functioning) [5]. As measured using WHODAS-12, we viewed disability and functioning as opposite ends of the same spectrum; high disability represents low functioning (or limitations in functioning) and low disability represents high functioning. In chronic LBP, WHODAS-36 has adequate content validity, structural validity, internal consistency, and reliability, and WHODAS-12 has adequate structural validity and internal consistency [6]. Scores from the short and full versions of WHODAS 2.0 are highly correlated [11, 12]. If questionnaires were missing only one item, WHODAS-12 scores based on a sum of the non-missing items, rescaled to maintain range from 0 to 48 can be used according to the WHODAS 2.0 manual [5]. We applied this rule when the work item was missing, as it was the most frequently missing item.

### Anchor measures and minimal important difference

We used change in the SF-36 PF in study 1 and change in the ODI in study 2 as anchor measures to identify subjects experiencing small but important improvements in functioning over time. The SF-36 questionnaire is a generic 36-item questionnaire for measuring health-related quality of life [13]. It includes eight individual dimensions, including physical functioning (SF-36 PF). SF-36 PF is composed of 10 items with a 3-point rating scale (higher scores indicate better health status). The SF-36 questionnaire has adequate validity, reliability, and responsiveness in persons with musculoskeletal conditions [14–17]. The ODI is a questionnaire that measures functional limitations specific to back pain [18]. The questionnaire has 10 questions and is scored 0-100 (higher scores indicate higher disability). The ODI has adequate validity and reliability in persons with LBP [19, 20]. Informed by literature, a small but meaningful improvement was defined as MID of 5–16 on SF-36 PF [21–23] or 4–16 on the ODI [21, 24–29]; these MID ranges for SF-36 PF and ODI were identified based on previous literature focused on persons with LBP. For study 1, subjects whose scores on the SF-36 PF improved between 5 and 16 points inclusive were deemed to have experienced a small but important improvement in functioning and for study 2, subjects whose ODI scores improved between 4 and 16 points inclusive were deemed to have experienced a small but important improvement in functioning.

### Analysis

We estimated WHODAS-12 scores at baseline and follow-up utilizing individual scores of the stated WHODAS-36 items, which are specific questions common to both short and full versions. Among participants who improved and achieved the minimal important difference on SF-36 PF (MID 5–16) or ODI (MID 4–16), we calculated the corresponding mean change and 95% confidence interval on WHODAS-12. The analysis for this study was generated using SAS software v9.4. (Copyright © 2012–2018, SAS Institute Inc., Cary, NC, USA. SAS and all other SAS Institute Inc. product or service names are registered trademarks or trademarks of SAS Institute Inc., Cary, NC, USA.)

## Results

### Sample characteristics

Of 108 participants with chronic LBP in study 1 (Garin et al.) [9], 70 had SF-36 PF scores at baseline and follow-up, and thus are eligible for our study. Of those, 23 had improvements in SF-36 PF scores between 5 and 16 points, of which 18 had WHODAS-12 change scores. Of 92 participants with chronic LBP in study 2 (Cwirlej-Sozanska et al.) [10], 89 had ODI scores at baseline and follow-up to be eligible for our study. Of those, 62 had improvement in ODI scores between 4 and 16 points, of which 50 had WHODAS-12 change scores.

Among patients with chronic LBP (with baseline and follow-up SF-36 PF scores) in study 1, mean age was 54.1 years (SD 14.7) and 68.6% were female (Table 1). Most were married (59.4%) and had highest education attainment levels of completing primary or secondary school (47.0%), high school (19.7%), or college/university (31.8%). The mean baseline SF-36 PF score was 64.1 (SD 25.0) and mean baseline WHODAS-12 score was 15.6 (SD 5.6) (Additional File 2 A).

**Table 1:** Sample characteristics for study 1 subset with chronic LBP at baseline (n = 108) (Garin et al. [9])

	LBP Baseline Sample	Sample with SF36 PF Scores at Baseline & Follow-up
	N = 108	N = 70
Sex - N (%)		
Male	41 (38.0%)	22 (31.4%)
Female	67 (62.0%)	48 (68.6%)
Age - Mean (SD)	52.7 (14.6)	54.1 (14.7)
Marital Status - N (%)		
Never married	12 (11.3%)	9 (13.0%)
Married	62 (58.5%)	41 (59.4%)
Separated / Divorced	13 (12.3%)	9 (13.1%)
Widowed	12 (11.3%)	8 (11.6%)
Cohabiting	7 (6.6%)	2 (2.9%)
Education - N (%)		
Less than primary	2 (2.0%)	1 (1.5%)
Primary school complete	36 (35.0%)	17 (25.8%)
Secondary school complete	20 (19.4%)	14 (21.2%)
High school complete	18 (17.5%)	13 (19.7%)
College/university complete	26 (25.2%)	21 (31.8%)
Postgraduate degree complete	1 (1.0%)	0 (0.0%)
SF36 Bodily Pain – Mean (SD)^a^	36.0 (20.9)	38.5 (21.7)
SF36 Physical Function – Mean (SD)^a^	60.8 (25.2)	64.1 (25.0)
WHODAS-12 – Mean (SD)^b^	15.7 (6.4)	15.6 (5.6)

LBP – low back pain; SD – standard deviation; SF36 PF – Short-form-36 Physical Functioning; WHODAS-12 - World Health Organization Disability Assessment Schedule 2.0 12-item questionnaire

^a^SF-36 dimensions are scored from 0 to 100 (higher score indicates better health-related quality of life)

^b^WHODAS-12 is scored from 0 to 48 (higher score indicates greater limitations in functioning)

Among patients with chronic LBP (with baseline and follow-up ODI scores) in study 2, mean age was 65.5 years (SD 11.5), and 60.7% were female (Table 2). More than half (52.8%) were from the countryside, and 61.8% had secondary or higher education. Mean baseline ODI score was 29.4 (SD 6.3) and mean baseline WHODAS-12 score was 19.1 (SD 6.7) (Additional File 2B).

**Table 2:** Sample characteristics for study 2 at baseline (n = 92) (Cwirlej-Sozanska et al. [10])

	Baseline Sample (N = 92)	Sample with ODI at Baseline & Follow-up (N = 89)
Sex - N (%)		
Male	35 (38.0%)	35 (39.3%)
Female	57 (62.0%)	54 (60.7%)
Age – Mean (SD)	66.0 (11.6)	65.5 (11.5)
Place or Residence		
City	44 (47.8%)	42 (47.2%)
Countryside	48 (52.2%)	47 (52.8%)
Education - N (%)		
Primary	18 (19.6%)	17 (19.1%)
Vocational	17 (18.5%)	17 (19.1%)
Secondary	45 (48.9%)	43 (48.3%)
Higher education	12 (13.0%)	12 (13.5%)
Pain (VAS) – Mean (SD)^a^	5.77 (1.31)	5.75 (1.31)
ODI – Mean (SD)^b^	29.6 (6.4)	29.4 (6.3)
WHODAS-12 – Mean (SD)^c^	19.3 (6.8)	19.1 (6.7)

ODI – Oswestry Disability Index; SD – standard deviation; VAS – Visual Analogue Scale; WHODAS-12 – World Health Organization Disability Assessment Schedule 2.0 12-item questionnaire

^a^VAS is scored from 0 to 10 (higher score indicates higher pain intensity)

^b^ODI is scored from 0 to 100 (higher score indicates higher disability)

^c^WHODAS-12 is scored from 0 to 48 (higher score indicates greater limitations in functioning)

### Minimal important difference of WHODAS-12

Of 70 eligible participants in study 1 (Garin in et al.) [9], 18 achieved a small meaningful improvement based on SF-36 PF and had WHODAS-12 change scores in the data. The corresponding mean WHODAS-12 change score was − 3.22/48 (95% CI -4.79 to -1.64; minimum − 10.00, maximum 2.18). Of 89 eligible participants in study 2 (Cwirlej-Sozanska et al.) [10], 50 achieved a small meaningful improvement based on ODI and had WHODAS-12 change scores in the data. The corresponding mean WHODAS-12 change score was − 5.99/48 (95% CI − 7.20 to -4.79; minimum − 16.36, maximum 2.18).

## Discussion

We estimated an MID of WHODAS-12 of -3.22/48 (95% CI -4.79 to -1.64) from one study and − 5.99/48 (95% CI -7.20 to -4.79) from another study in adults with chronic LBP. The MIDs of WHODAS-12 were calculated using an anchor-based approach by considering the achievement of MID threshold improvements on SF-36 PF and ODI. Our study advances knowledge in this area by providing MID estimates for WHODAS-12 specific to persons with chronic LBP.

Our findings on MID for WHODAS-12 in persons with chronic LBP are similar to those of the previous study in Finland in patients with musculoskeletal conditions (including LBP) [7]. Katajapuu et al. estimated MIDs of WHODAS-12 as 3.09/48 (using 0.33xSD), 3.10/48 (using standard error of the mean), and 4.68/48 (using 0.5xSD), calculated using distribution-based methods [7]. Our findings are based on an anchor-based approach using WHODAS-12 change scores to compute the MID specific to chronic LBP instead of distribution-based methods, which are based on baseline measures of WHODAS-12 only. Anchor-based approaches take into account the patient perspective of the minimal difference that is clinically important to them and also utilize change in the WHODAS-12 measured at two points in time. This is an added strength to our findings to advance knowledge in this area, as anchor-based methods are recommended based on COSMIN [8]; notably, further to anchor-based methods, triangulation of multiple methods (based on consensus, anchor-based, and distribution approaches) may be most informative for estimating the MID for WHODAS-12 [30].

Our findings of MIDs − 3.22 and − 5.99 suggest variability in this threshold of important benefit. This is aligned with MIDs estimated for other outcome measures, such as those used in our study. This includes MIDs ranging from 5 to 16 for SF-36 PF [21–23] and MIDs ranging from 4 to 16 for ODI [21, 24–29] in persons with LBP as informed by literature. Some variability in MIDs (e.g., MIDs in WHODAS, SF-36, ODI, or other instruments) is attributable to context and patient characteristics, such as time periods of change, severity at baseline, and anchors used [31]. Informed by previous literature on methodology and credibility of estimating MIDs [32, 33], the MID of -5.99/48 calculated from study 2 (Cwirlej-Sozanska et al.) may be the more robust estimate for two main reasons. The anchor of ODI in study 2 more closely reflects the constructs captured in WHODAS. The ODI focuses on LBP-related limitations in functioning, while the WHODAS captures limitations in functioning more broadly (i.e., not specific to LBP). Although there is overlap, the SF-36 focuses on health-related quality of life, which is a different construct from limitations in functioning. In addition, the sample in study 2 is larger and has less missing data, allowing for more precision of the WHODAS-12 MID estimate. When using this questionnaire to measure functioning, it is noted that there are potential floor effects with WHODAS-12 summary scores. In a study by Katajapuu et al., a significant floor effect (set at > 15%) was observed for WHODAS-12 summary scores using simple scoring, but no ceiling effects were observed in persons with chronic musculoskeletal conditions [34].

Our findings have potential implications for measuring functioning for chronic LBP related to rehabilitation services. To determine whether the delivery of rehabilitation is meaningful for patients, we need to assess whether rehabilitation achieves a threshold of important benefit. Health care providers can use WHODAS-12 to measure functioning and assess for achieving MID in patients. This helps to guide management and effective rehabilitation care using WHODAS-12 as an outcome measure. Moreover, our findings may inform sample size considerations for future RCTs focused on measuring functioning in samples with chronic LBP. Multiple methods may be used to inform the estimation of MID [30], so our findings can be one part of broader considerations in calculating sample size in these future studies.

### Strengths and limitations

Our study has strengths. We analyzed data from two cohort studies conducted in Europe to compute two estimates of MID for WHODAS-12 in adults with chronic LBP. Notably, we used an anchor-based approach to account for those who achieved a minimal difference that was clinically important to patients on SF-36 PF or ODI. We selected MIDs for SF-36 PF and ODI in persons with LBP based on previous literature. In addition, the questionnaires WHODAS 2.0, SF-36 Health Survey, and ODI have adequate validity and reliability in persons with back pain or musculoskeletal conditions [6, 14–17, 19, 20].

Our study has limitations. First, there is potential selection bias due to missing data. In the study by Garin et al [9], 40 out of 118 participants were missing data on SF-36 PF. In the study by Cwirlej-Sozanska et al [10], 3 out of 92 participants were missing data on the ODI. Our findings are limited by small samples of the two studies and missing data, which leads to imprecision of the MID estimates that we calculated. In study 1, the participants who stayed in versus dropped out are different across various characteristics; those who dropped out tended to have the following characteristics: male, lived alone, smoker, younger, higher levels of disability and pain, lower physical function/component of health-related quality of life, and higher mental component of health-related quality of life [9] (see Additional File 3). These demonstrate that the data is not missing completely at random. While there is no way to be sure, these differences lead us to be wary of assuming that they are missing at random. The reasons for these missing data in the original cohort study by Garin et al. are not known. Second, it is important to consider that MIDs may vary by contexts to inform the generalizability of our findings. Literature suggests that MIDs may vary depending on characteristics of the study population (which can include baseline severity on measure of interest), duration of follow-up and type of intervention [31]. Therefore, knowledge users looking to use MID estimates for WHODAS-12 in persons with chronic LBP should consider whether these factors underlying our MID estimates are similar to the contexts in which they would like to apply the WHODAS MID estimates.

## Conclusion

Using an anchor-based approach, the MID of WHODAS-12 is estimated at -3.22/48 (95% CI -4.79 to -1.64) or -5.99/48 (95% CI − 7.20 to -4.79) in persons with chronic LBP. These MID values inform the utility of WHODAS-12 in measuring functioning to determine whether rehabilitation or other health services achieve a minimal difference that is meaningful to the patient. Health care providers can consider using these MID values with WHODAS-12 as an outcome measure to assess whether rehabilitation is providing important benefits to patients. Overall, findings have implications for the measurement of important benefits in functioning levels related to rehabilitation services for chronic LBP.

### Electronic supplementary material

Below is the link to the electronic supplementary material.

Supplementary Material 1

## Data Availability

The datasets used and/or analysed during the current study may be available from the corresponding author of the original studies on reasonable request.

## Abbreviations

CI: Confidence interval
COSMIN: COnsensus–based Standards for the selection of health Measurement Instruments
LBP: Low back pain
MID: Minimal important difference
ODI: Oswestry Disability Index
SD: Standard deviation
SF-36 PF: Short Form–36 Physical Functioning
WHO: World Health Organization
WHODAS: World Health Organization Disability Assessment Schedule
