# Supplementary material for: Minimal important difference of the 12-item World Health Organization Disability Assessment Schedule (WHODAS) 2.0 in persons with chronic low back pain
Source: Chiropr Man Therap. 2023 Dec 5;31:49. doi: 10.1186/s12998-023-00521-0 (PMC10696846; doi:10.1186/s12998-023-00521-0)
Supplement: Supplementary file 1 — Supplementary Material 1 [file 12998_2023_521_MOESM1_ESM.pdf]

SUPPLEMENTARY FILE

Title: Minimal important difference of the 12-item World Health Organization Disability Assessment Schedule (WHODAS) 2.0 in persons with chronic low back pain

Authors: Jessica J. Wong; Sheilah Hogg-Johnson; Wouter De Groote; Agnieszka Ćwirlej-Sozańska; Olatz Garin, Montse Ferrer, Àngels Pont Acuña, Pierre Côté

Corresponding author: Jessica Wong, Institute for Disability and Rehabilitation Research, Ontario Tech University, 2000 Simcoe Street North, Oshawa, Ontario, Canada L1G 0C5. E-mail address: jessica.wong@ontariotechu.ca

Additional File 1. World Health Organization Disability Assessment Schedule 2.0 12 item (WHODAS)-12 (short version with 12 questions)[1]

**PLEASE NOTE: When scoring WHODAS, the following numbers are assigned to responses:**	
	**0 = No Difficulty**
	**1 = Mild Difficulty**
	**2 = Moderate Difficulty**
	**3 = Severe Difficulty**
	**4 = Extreme Difficulty or Cannot Do**
S1	Standing for long periods such as 30 minutes?
S2	Taking care of your household responsibilities?
S3	Learning a new task, for example, learning how to get to a new place?
S4	How much of a problem did you have in joining in community activities (for example, festivities, religious or other activities) in the same way as anyone else can?
S5	How much have you been emotionally affected by your health problems?
S6	Concentrating on doing something for ten minutes?
S7	Walking a long distance such as a kilometre [or equivalent]?
S8	Washing your whole body?
S9	Getting dressed?
S10	Dealing with people you do not know?
S11	Maintaining a friendship?
S12	Your day-to-day work/school?

Additional File 2. Histograms of the WHODAS 2.0 12 Item Scores from Baseline for the Two Study Samples

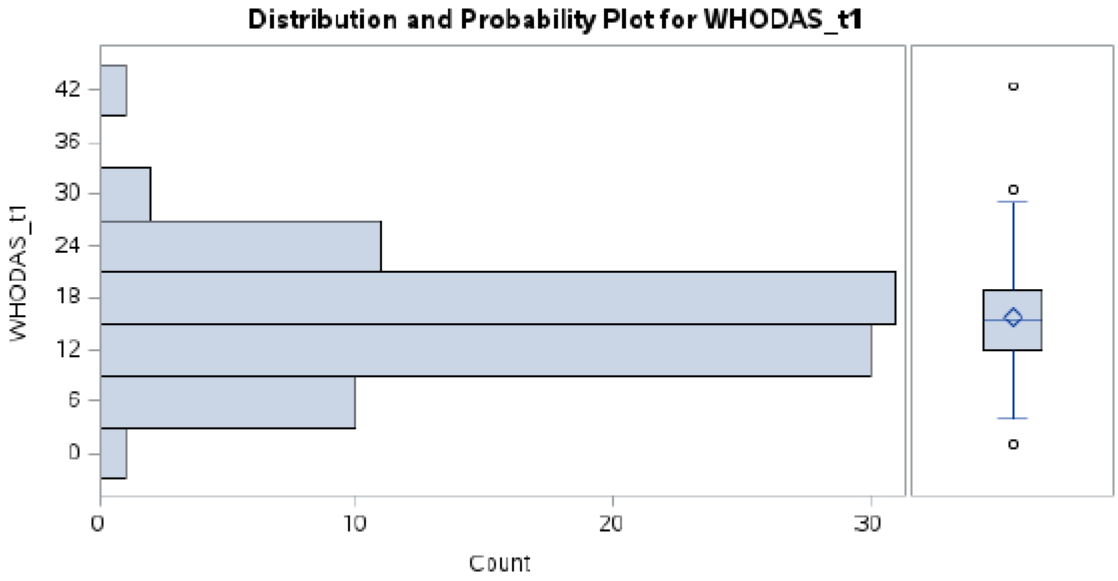

A. Baseline distribution of WHODAS 2.0 12 item scores from study of Garin et al (N=108 with cLBP)

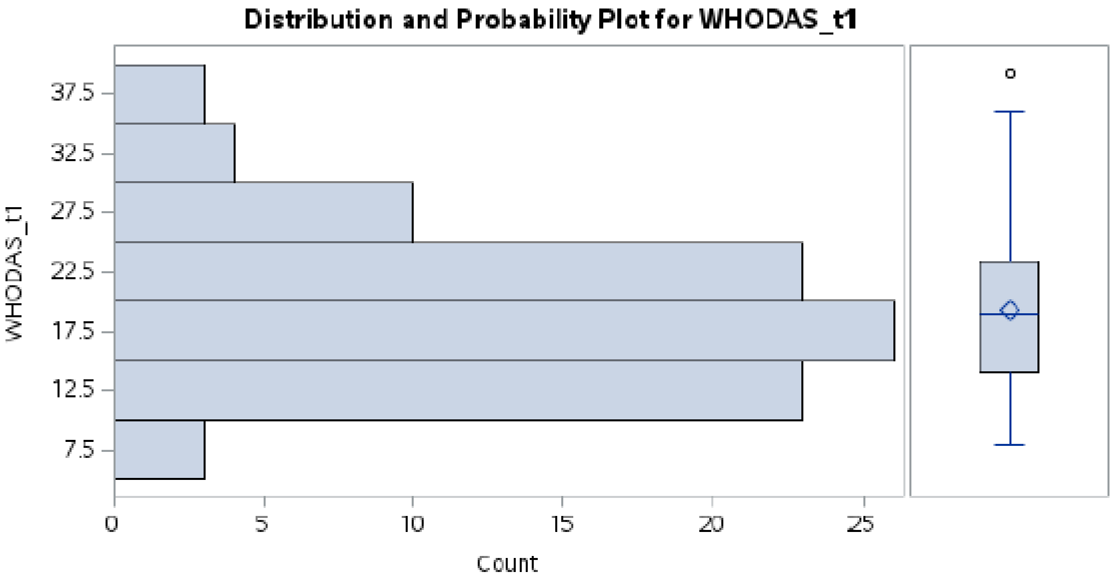

B. Baseline distribution of WHODAS 2.0 12 item scores from study of Ćwirlej-Sozańska et al (N=92)

Additional File 3. Comparison of characteristics among participants who stayed in versus dropped out in Garin et al study[2]

	Dropout (N=38)	Included (N=70)
	N (%)	N (%)
Sex - female	19 (50.0%)	48 (68.6%)
National - yes	30 (81.1%)	67 (95.7%)
Living Alone - yes	11 (29.0%)	15 (21.4%)
Work Status		
Employee	12 (32.4%)	23 (33.3%)
Self-employed	1 (2.7%)	2 (2.9%)
Employer	3 (8.1%)	8 (11.6%)
Not working for pay	21 (56.8%)	36 (52.2%)
Smoking		
Daily	10 (26.3%)	17 (24.3%)
Not daily	4 (10.5%)	0 (0.0%)
Not at all	24 (63.2%)	53 (75.7%)
Alcohol Consumption - yes	30 (79.0%)	51 (76.1%)
LBP Severity		
Mild	13 (35.1%)	24 (35.8%)
Moderate	19 (51.4%)	24 (36.8%)
Severe	5 (13.5%)	19 (28.4%)
	Mean (SD)	Mean (SD)
Age	50.2 (14.3)	54.1 (14.7)
Years of School	12.9 (3.7)	13.3 (3.7)
WHODAS 2.0 12-item Summary baseline	16.1 (8.2)	15.6 (5.6)
WHODAS 2.0 36-item Dimensions		
Understanding & Communicating	22.5 (21.9)	17.1 (16.8)
Getting Around	38.9 (21.3)	30.9 (23.1)
Self Care	18.6 (20.5)	8.6 (13.5)
Getting Along with People	18.1 (20.5)	11.1 (15.8)
Life Activities Household	46.0 (28.3)	34.5 (28.1)
Life Activities Work/School	41.1 (32.0)	20.9 (11.5)
Participation in Society	37.5 (21.2)	26.0 (21.8)
SF36 Bodily Pain baseline	31.3 (18.8)	38.5 (21.7)
SF36 Physical Function baseline	52.2 (24.3)	64.1 (25.0)
SF36 Physical Component Score baseline	32.9 (8.1)	39.2 (10.4)
SF36 Mental Component Score baseline	46.4 (12.2)	35.9 (11.5)

*Those who dropped out tended to have the following characteristics: male, lived alone, smoker, younger, higher levels of disability and pain, lower physical function/component of health-related quality of life, higher mental component of health-related quality of life

References

1. World Health Organization. Measuring health and disability: manual for World Health Organization (WHO) Disability Assessment Schedule 2.0 (WHODAS 2.0). 2012. Available at: https://www.who.int/standards/classifications/international-classification-of-functioning-disability-and-health/who-disability-assessment-schedule (accessed Apr 23 2021).
2. Garin O, Ayuso-Mateos JL, Almansa J, Nieto M, Chatterji S, Vilagut G, et al. Validation of the "World Health Organization Disability Assessment Schedule, WHODAS-2" in patients with chronic diseases. Health Qual Life Outcomes. 2010;8:51.
